# Pulmonary nodules detection based on multi-scale attention networks

**DOI:** 10.1038/s41598-022-05372-y

**Published:** 2022-01-27

**Authors:** Hui Zhang, Yanjun Peng, Yanfei Guo

**Affiliations:** 1 College of Computer Science and Engineering, Shandong University of Science and Technology, Qingdao, 266590 Shandong China; 2 Shandong Province Key Laboratory of Wisdom Mining Information Technology, Shandong University of Science and Technology, Qingdao, 266590 Shandong China

**Keywords:** Cancer screening, Lung cancer, Image processing, Network models

## Abstract

Pulmonary nodules are the main manifestation of early lung cancer. Therefore, accurate detection of nodules in CT images is vital for lung cancer diagnosis. A 3D automatic detection system of pulmonary nodules based on multi-scale attention networks is proposed in this paper to use multi-scale features of nodules and avoid network over-fitting problems. The system consists of two parts, nodule candidate detection (determining the locations of candidate nodules), false positive reduction (minimizing the number of false positive nodules). Specifically, with Res2Net structure, using pre-activation operation and convolutional quadruplet attention module, the 3D multi-scale attention block is designed. It makes full use of multi-scale information of pulmonary nodules by extracting multi-scale features at a granular level and alleviates over-fitting by pre-activation. The U-Net-like encoder-decoder structure is combined with multi-scale attention blocks as the backbone network of Faster R-CNN for detection of candidate nodules. Then a 3D deep convolutional neural network based on multi-scale attention blocks is designed for false positive reduction. The extensive experiments on LUNA16 and TianChi competition datasets demonstrate that the proposed approach can effectively improve the detection sensitivity and control the number of false positive nodules, which has clinical application value.

## Introduction

In recent years, lung cancer has become a major disease threatening human life and health. According to the global cancer statistics in 2018, the incidence and mortality of lung cancer rank first among all cancers in the world^1^. The five-year survival rate for lung cancer patients is only about 16%. Pulmonary nodules are the main manifestation of early lung cancer. If the nodules are detected in the early diagnosis of lung cancer, the five-year survival rate of patients will increase to 70%^2^. Low-dose Computed Tomography (CT) has demonstrated to be an effective tool for pulmonary nodules detection. However, the large number of CT images increases the workload of physicians, and thus leads to an increase in misdiagnosis. Therefore, the use of advanced computer-aided diagnosis (CAD) technology for lesion diagnosis has always been a hot field of medical image processing research. With the development of deep learning, especially the successful application of convolutional neural network (CNN) in the field of medical image processing^3,4^, employing CNN for automatic detection of pulmonary nodules has reported promising results in improving detection sensitivity and reducing false positive rate.

Xie et al.^5^ used two regional proposal networks (RPN)^6^ and a deconvolutional layer to adjust 2D Faster R-CNN^7^ for candidate nodules detection and then trained different slices with three 2D models for false positive reduction. They reported an average sensitivity at 7 false positive numbers (0.125, 0.25, 0.5, 1, 2, 4, 8) of 0.790 on the LUNA16 dataset^8^. Gu et al.^9^ presented a pulmonary nodule detection model based on 2D deformable convolution to solve the problems of different sizes and irregular shapes of nodules. The average sensitivity on the LIDC-IDRI dataset^10^ is 0.827. Yuan et al.^11^ designed a deep residual CNN for false positive reduction, using deformable convolution to adaptively reflect different spatial information, and resulting in an average sensitivity of 0.835 on LUNA16 dataset. Although the 2D methods had the advantages of small storage space and short training time during the training phase, they were still limited in making full use of the 3D spatial information in CT images. Lately, Dou et al.^12^ proposed a 3D fully convolutional network (FCN)^13^ to detect candidate nodules in the LUNA16 dataset and reported an average sensitivity of 0.839. Then they designed a hybrid-loss residual network for false positive reduction with a sensitivity of 0.905 at 1 false positive per scan. Zhu et al.^14^ used a 3D Faster R-CNN with dual path blocks^15^ and a U-net-like encoder-decoder structure^16^ for candidate nodule detection, reaching an average sensitivity of 0. 842 on the LUNA16 dataset. Dou et al.^17^ presented a novel multi-level contextual 3D CNN framework, by integrating a set of 3D CNNS with different sizes of receptive fields to achieve false positive reduction and reached an average sensitivity of 0.827 on the LUNA16 dataset. Despite the effectiveness of 3D models in improving the sensitivity of nodule detection, they still have some problems. Firstly, most existing methods exploit the multi-scale features from a layer level of the CNN model to detect nodules. However, the expression of multi-scale functions at a layer level is limited. Secondly, it will lead to over-fitting and reduce the detection sensitivity if the features extracted by the 3D CNN are too complex and comprehensive.

Peng et al.^18^ proposed a 3D multi-scale pulmonary nodule detection method based on deep CNNs. By embedding squeeze-and-excitation unit^19^ into Res2Net^20^ residual blocks, they designed a multi-scale attention (MSA) structure. Then based on this module, a 3D nodule candidate detection network and a false positive reduction network were created. The model achieved an average sensitivity of 0.923 on the LUNA16 dataset. Although the model can extract multi-scale features of nodules, we believe that there are still some unresolved issues in this paper. On the one hand, the paper does not provide a solution for alleviating the network overfitting problem. On the other hand, it is difficult to illustrate the generalization performance of the model using only the LUNA16 dataset. Finally, there is a lack of regional segmentation of the original CT image in the data pre-processing. To make full use of multi-scale information of nodules and alleviate the network overfitting, we propose an automatic pulmonary nodule detection system based on MSA networks. Instead of using a squeeze-and-excitation unit that requires a certain number of learnable parameters, this paper embeds an almost parameter-free convolutional quadruplet attention module (CQAM) in Res2Net. It not only reduces the number of parameters but also improves the model detection performance by capturing cross-dimension interaction of feature maps. Then we creatively add the pre-activation operation^21^ to Res2Net to alleviate the over-fitting problem. In the end, this paper tests the model generalization performance on the TianChi dataset and implements the region segmentation of CT images.

The contributions of this paper are as follows: (a) This paper proposes a 3D MSA block that can extract multi-scale features at the granularity level through the Res2Net for the full utilization of nodule multi-scale information. It effectively alleviates network over-fitting by pre-activation and further improves the detection sensitivity by CQAM. (b) To fully exploit the 3D CT images, this paper proposes a 3D Faster R-CNN based on 3D MSA blocks and a U-net-like encoder-decoder structure to automatically detect pulmonary nodules, and a 3D deep multi-scale attention networks to reduce false positive numbers. (c) The proposed system achieves a CPM score of 0.927 on the LUNA16 dataset, which indicates that the model has excellent performance for accurate nodule detection. In the candidate nodule detection stage, the CPM score of 0.679 on the TianChi dataset shows that the model also has good generalization ability.

The rest of this paper is organized as follows. In “Method" section introduces the framework of the proposed approach, in “Experiment and results" section presents the experimental details and analysis of the results. Finally, in “Conclusion and discussion” section gives the conclusion and discussion of the work.

## Method

The method was performed in accordance with the relevant guidelines and regulations, with informed consents obtained from all subjects. The LUNA16 dataset (https://luna16.grand-challenge.org/Data/ established by the NIH and NCI of the United States) is used to train and test the proposed model. This dataset is freely available to browse, download, and use for commercial, scientific and educational purposes as outlined in the Creative Commons Attribution 4.0 International License. The TianChi dataset (https://tianchi.aliyun.com/competition/entrance/231601/information) is adopted to evaluate the model generalization performance, which is the competition dataset of the TianChi Medical AI Competition [Season One] co-sponsored by Alibaba Cloud, Intel, and LinkDoc. The dataset is authorized by the partner hospital of the competition and can be downloaded for free online.

**Figure 1:**
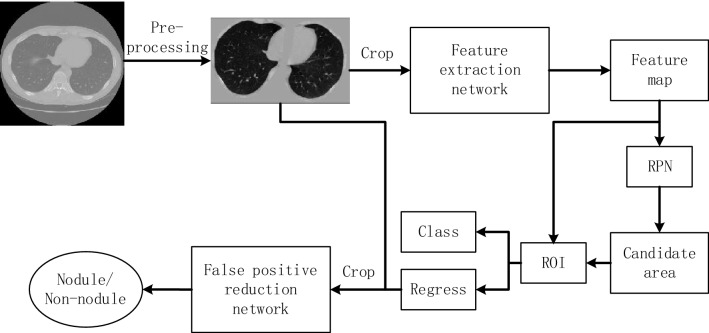
Automatic pulmonary nodule detection system based on MSA convolutional neural network.

Automatic detection of pulmonary nodules is a target detection task that inputs CT images and outputs the position of nodules, which aiming to detect candidate nodules with high sensitivity and restrict the average number of false positives per scan. To achieve this goal, this paper proposes a pulmonary nodule detection system based on multi-scale attention networks, the structure of which is shown in Fig. 1. Firstly, the lung parenchyma images are obtained by pre-processing the original CT image. Secondly, the cropped lung parenchymal images are input into the feature extraction network for extracting features. And then the resulting feature map is subjected to RPN and RoI operations to obtain the positions and probability of candidate nodules. Finally, the false positive cases in the candidate nodules are removed by the false positive reduction network to achieve the final result.

### Multi-scale attention block

**Figure 2:**
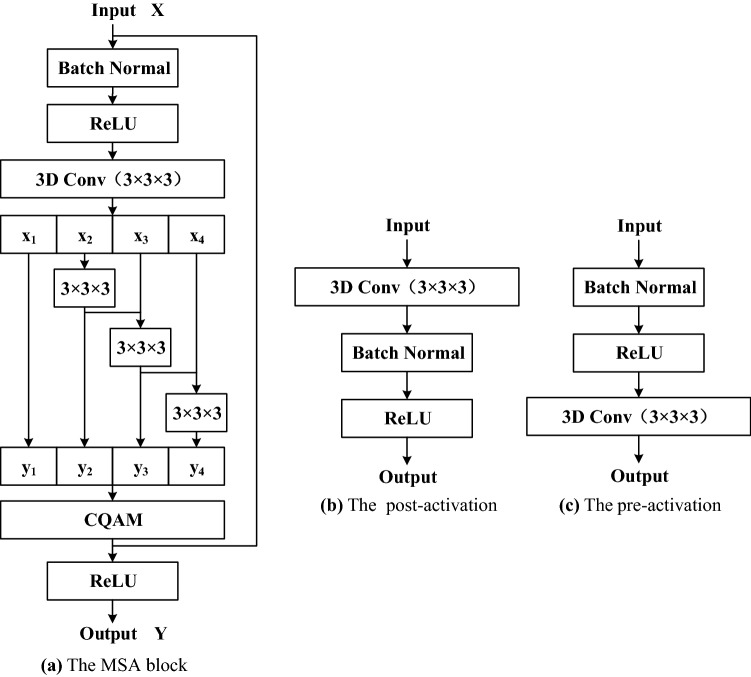
The structures of the MSA block, post-activation and pre-activation.

The MSA block is composed of Res2Net module, pre-activation unit and CQAM. Its structure is shown in Fig. 2. The Res2Net structure represents multi-scale features at a granular level, which can fully exploit multi-scale information compared with the layer-wise manner. The pre-activation operation can effectively alleviate over-fitting phenomenon. And CQAM computers attention weights by capturing cross-dimension interaction using a four-branch structure to emphasize useful information and thus improve the pulmonary nodule detection sensitivity.

In the MSA block, the post-activation is replaced by pre-activation that consists of Batch Normalization (BN)^22^ and Rectified Linear Unit (ReLU)^23^. Although the post-activation method can standardize the signal, it will be quickly incorporated into the shortcut connection, and the combined signal is not standard. This non-standard signal is input into the next convolution layer, which causes the extracted nodule features to be complex, while the complex features will lead to serious over-fitting. Therefore, pre-activation unit is added to the front of each convolution layer to perform BN and ReLU activation on the pulmonary nodule that input to the convolution layer. BN algorithm can reduce the complexity of nodule features by standardizing input signals. ReLU activation operation can increase the nonlinear relationship between the convolution layers to make the neurons in the convolution neural network have sparse activation, which is conducive to the model to better mine the nodule-related features. Therefore, the use of pre-activation unit ensures that the input of each convolution layer is standardized and activated, which effectively alleviates the over-fitting problem.

The MSA block first uses the pre-activated operation and a 3 × 3 × 3 standard convolution layer to extract features from the input tensor, and the output feature is evenly split into 4 feature map subsets (denoted as $x_{1}$, $x_{2}$, $x_{3}$, $x_{4}$ in Fig. 2) according to the channel dimension. Each feature subset has the same spatial size. Except for $x_{1}$, each $x_{i}$ has a corresponding 3 × 3 × 3 small filter, denoted by $K_{i}$. The feature subset $x_{i}$ is added with the output of $K_{i - 1}$, and then fed into $K_{i}$ for convolution calculation to obtain the output $y_{i}$. The calculation formula of $y_{i}$ is as follows:

1 $$y_{i} = \left\{ {\begin{array}{*{20}l} {x_{i} ,i = 1} \\ {K_{i} \left( {x_{i} } \right),i = 2} \\ {K_{i} \left( {y_{i - 1} + x_{i} } \right),3 \le i \le 4} \\ \end{array} } \right.$$

Finally, $y_{1}$, $y_{2}$,$y_{3}$, $y_{4}$ are concatenated according to the channel dimensions. In MSA block, the 3 × 3 × 3 large filter which have n channels is replaced with a set of 3 × 3 × 3 smaller filters of m channels (*n* = m × s, s is the number of small filters). These smaller filters are connected in a hierarchical residual-like style to increase the number of scales that the output features can represent, and thus realizing the use of multi-scale features of lung nodules at a finer-grained level. In addition, omitting the convolution for $x_{1}$ not only reduces the number of parameters, but also reuses the features. Finally, the use of split and concatenation allows convolution to process features more efficiently.

**Figure 3:**
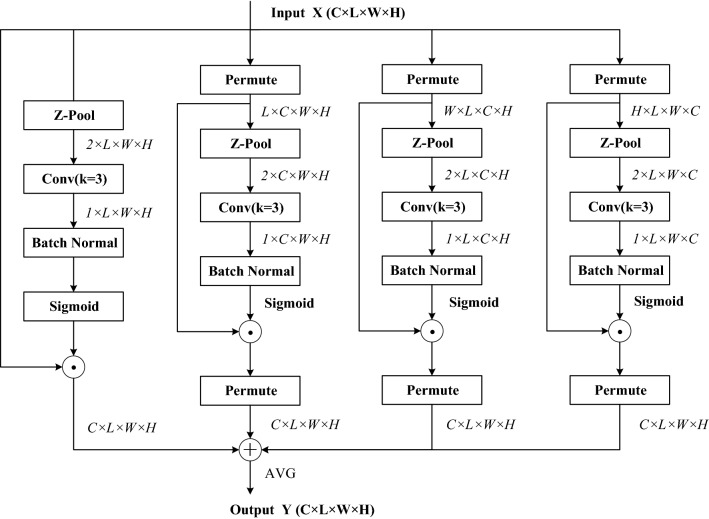
The structure of CQAM.

The structure of CQAM is shown in Fig. 3. This module is based on the convolutional triplet attention module (CTAM)^24^ which designed for 2D input images, we change the original three-branch structure to four-branch for processing 3D input data. The CQAM captures the cross-dimension interaction between the channel dimension and spatial dimension through four branches, computes attention weights to provide rich feature representations, and emphasizes the feature information useful for pulmonary nodule detection. Therefore, it can greatly improve the sensitivity of nodule detection. Moreover, unlike squeeze-and-excitation networks (SENet) that require a certain number of learnable parameters, it requires almost no parameters. The first branch in Fig. 3 is used to capture spatial dependencies. Firstly, the number of channels of the input tensor $x \in R^{C \times L \times W \times H}$ is reduced to two by the Z-Pool pooling operation. Then the reduced tensor $x_{1} \in R^{2 \times L \times W \times H}$ is fed into the convolutional layer, batch normalization layer, and sigmoid activation layer in turn to obtain the attention weights $w_{1} \in R^{1 \times L \times W \times H}$ . Finally, the output of this branch ${\text{y}}_{1}$ is generated by applying $w_{1}$ to the input feature. The remaining three branches adopt rotation operation to establish connections between the channel dimension and either one of the spatial dimensions. Especially, the second branch in Fig. 3 captures the cross-dimension interaction between the channel dimension and the length dimension. To achieve this, the given input $x \in R^{C \times L \times W \times H}$ is rotated to obtain the tensor $x_{2} \in R^{L \times C \times W \times H}$. The resultant attention weights $w_{2} \in R^{1 \times C \times W \times H}$ are generated after performing the same operation as the first branch on the rotated tensor $x_{2}$. Then $w_{2}$ is simply applied on $x_{2}$ and the result is subsequently rotated to get the output $y_{2}$. Finally, the results generated by four branches are aggregated by simple averaging to obtain the output of the module.

The mathematical formula of Z-Pool operation is expressed as Eq. (2). By concatenating the results of average pooling and max-pooling of the feature map, the zeroth channel dimension of the tensor is reduced to 2, which not only preserves the tensor rich representation but also reduces the depth and simplifies the calculation.

2 $$Z - Pool(x) = [Max\,Pool_{0d} (x),Avg\,Pool_{0d} (x)]$$

where $x$ is the input of Z-Pool operation, and *0d* means the 0th-dimension on which max and average pooling operations act.

### Nodule candidate detection

#### Architecture

Inspired by the success of Faster R-CNN on target detection, this paper uses Faster R-CNN as the basic detection framework. As shown in Fig. 1, the pre-processed lung parenchymal image is first input into a feature extraction network composed of MSA blocks and U-Net-like encoder-decoder structure. Then the extracted feature map is sent to the back-end detection network such as RPN to perform detection tasks, thereby obtaining the pulmonary nodule detection results.

**Figure 4:**
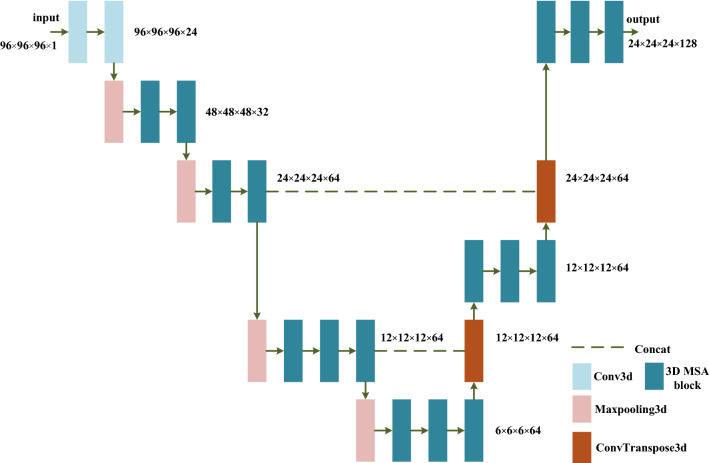
The structure of the feature extraction network based on 3D MSA blocks. The size of feature map is written in the form of (#length × #width × #height × #channel), such as (96 × 96 × 96 × 1).

With the encoder-decoder structure, the feature extraction network ensures the integrity of pulmonary nodule information by integrating high-level features and low-level features. Its structure is shown in Fig. 4. It is not feasible to input the entire image to the model due to GPU memory limitations. Therefore, the 96 × 96 × 96 nodules and non-nodules 3D data are randomly cropped from the lung parenchyma image as the input of the network. Before the first max-pooling, two 3 × 3 × 3 standard convolution layers are used to generate features. After that, the encoder sub-network composed of four max- pooling layers with a step size of 2 and 3D MSA blocks extracts nodule multi-scale features, reducing the size of the feature map to 6 × 6 × 6. In the decoder sub-network, the size of the feature map is first raised to 12 × 12 × 12 by 2 × 2 × 2 deconvolutional layer and concatenated with the lower features. Then it is restored to 24 × 24 × 24 after being processed by three MSA blocks and a deconvolutional layer and the feature map subsequently concatenated with the corresponding layers in the encoder network. Finally, the output feature map with a dimension of 24 × 24 × 24 is obtained through three MSA blocks. The resulting feature map is fed into the RPN for ROI classification and regression.

#### Loss function

The loss function in this paper consists of classification loss and regression loss. According to the size distribution of pulmonary nodules, the sizes of network candidate area are set to 5 × 5 × 5, 10 × 10 × 10 and 20 × 20 × 20. The intersection over union (IoU) in the target detection task is calculated to determine whether the candidate area is a target in the nodule detection task, which is the pulmonary nodule in the task of this paper. If the candidate area overlaps a target nodule with an IoU higher than a threshold of 0.5, we consider this candidate area contains nodules and mark it as a positive label ($p_{i}^{*}$ = 1). In contrast, if the candidate area has IoU with all target nodules less than 0.02, it means there is no pulmonary nodules in this area, and thus it is regarded as a negative label ($p_{i}^{*}$ = 0). All other candidate regions are ignored during training and do not contribute to the loss. And notice that only samples with positive labels are considered for regression loss. The multi-task loss function of each marked candidate nodule is defined as follows:

3 $$L\left( {p_{i} ,t_{i} } \right) = \lambda L_{cls} \left( {p_{i} ,p_{i}^{*} } \right) + p_{i}^{*} L_{reg} \left( {t_{i} ,t_{i}^{*} } \right)$$

where $i$ is the index of the current candidate box. The hyperparameter $\lambda$ for balancing the classification loss and regression loss is set to 0.5. The classification loss $L_{cls}$ uses the binary cross-entropy loss function, and the regression loss $L_{reg}$ uses the smooth $L1$ loss function, which are defined as follows:

4 $$L_{cls} \left( {p_{i} ,p_{i}^{*} } \right) = p_{i}^{*} \log \left( {p_{i} } \right) + \left( {1 - p_{i}^{*} } \right)\log \left( {1 - p_{i} } \right)$$

5 $$L_{reg} \left( {t_{i} ,t_{i}^{*} } \right) = \left\{ {\begin{array}{*{20}l} {0.5\left( {t_{i} - t_{i}^{*} } \right)^{2} \times \frac{1}{{\sigma^{2} }}\,\,\,\,if \, \left| {t_{i} - t_{i}^{*} } \right| < \frac{1}{{\sigma^{2} }}} \\ {\left| {t_{i} - t_{i}^{*} } \right| - 0.5,\,\,\,\,\,otherwise} \\ \end{array} } \right.$$

where $p_{i}$ and $p_{i}^{*}$ represent the predicted probability and classification label of the candidate region, respectively. The value of $\sigma$ is set to 1. And $t_{i}$ is the predicted relative coordinates for the candidate area, $t_{i}^{*}$ is the target nodule position. They are defined as follows:

6 $$t_{i} = \left( {\frac{{x - x_{\alpha } }}{{d_{\alpha } }},\frac{{y - y_{\alpha } }}{{d_{\alpha } }},\frac{{z - z_{\alpha } }}{{d_{\alpha } }},\log \left( {\frac{d}{{d_{\alpha } }}} \right)} \right)$$

7 $$t_{i}^{*} = \left( {\frac{{x^{*} - x_{\alpha } }}{{d_{\alpha } }},\frac{{y^{*} - y_{\alpha } }}{{d_{\alpha } }},\frac{{z^{*} - z_{\alpha } }}{{d_{\alpha } }},\log \left( {\frac{{d^{*} }}{{d_{\alpha } }}} \right)} \right)$$

where $\left( {x,y,z,d} \right)$ are the coordinates and diameter of the predicted nodule in the original space, $\left( {x_{\alpha } ,y_{\alpha } ,z_{\alpha } ,d_{\alpha } } \right)$ are the coordinates and size of the current candidate box and $\left( {x^{*} ,y^{*} ,z^{*} ,d^{*} } \right)$ are the coordinates and diameter of the real nodule in the original space.

### False positive reduction

In the previous candidate nodule detection stage, numerous candidate nodules were generated, in which there are still some false positive cases. To distinguish true nodules from highly similar false positive samples and improve the detection accuracy, a 3D deep CNN based on MSA blocks is constructed to further classify the candidate nodules.

**Figure 5:**
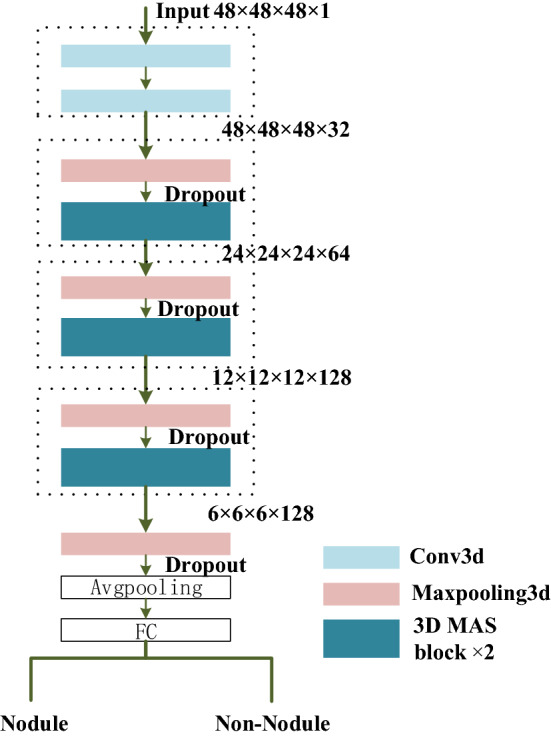
The structures of false positive reduction network. The size of feature map is written in the form of (#length × #width × #height × #channel), such as (48 × 48 × 48 × 1).

As shown in Fig. 5, the network consists of convolution layers, maximum pooling layers, and MSA blocks. To reduce the amount of calculation, a 48 × 48 × 48 data cube is cropped based on the candidate nodule coordinates and input into the network. This size can ensure that the nodule is completely contained in the cube. The input image is first fed into 2 convolution layers with kernel size of 3. Then it goes through three sets of max-pooling layers and MSA blocks for down-sampling and feature extraction. Finally, the average pooling layer and fully connected layer after a max-pooling layer classify the candidate nodules as true nodules or false positive ones. In addition, a dropout layer^25^ is used to enhance the generalization ability of the model and the binary cross-entropy loss function is used for optimization.

## Experiment and results

### Datasets and pre‑processing

This paper uses the LUNA16 dataset to train the network, which comes from the LIDC-IDRI public dataset. LUNA16 dataset contains 888 CT scans with a slice thickness smaller than 2.5 mm, and a total of 1186 lung nodules that marked by at least three radiologists. The position coordinates and diameter information of each nodule are given in an attached csv file. The nodules diameter ranges from 3.0 mm to 28.3 mm and its average size is 8.3 mm. Moreover, the LUNA16 dataset divides 888 CT images into 10 subsets for tenfold cross-validation.

To evaluate the generalization ability of the proposed method, the performance of the model was tested on a large-scale TianChi dataset, which includes 1000 CT scans from Chinese patients, a total of 1230 nodules marked by radiologists in position and diameter. Similar to the LUNA16 dataset, the original images of the TianChi dataset are also 3D images, which are composed of different numbers of 2D images of axial slices of the thoracic cavity. 200 CT scans from the validation set are adopted to evaluate our model.

**Figure 6:**
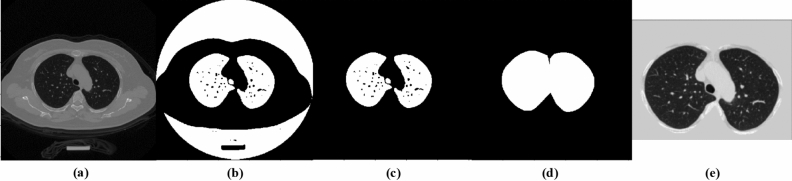
The pre-processing process of the TianChi dataset. (**a**) the raw image, (**b**) the binarized image, (**c**) the extracted original mask, (**d**) the final complete mask, (**e**) the lung parenchyma image.

To reduce the detection range of pulmonary nodules and facilitate the extraction of nodule features, it is necessary to preprocess the CT images and segment the lung parenchyma before model training. For LUNA16 dataset, firstly, the raw CT data is clipped into [− 1200, 600] according to the HU value of lung. Secondly, the image pixels are normalized to [0, 255]. Finally, the segmentation result given by LUNA16 is used to remove the background and obtain the lung parenchyma. Since the segmentation mask is not provided by the TianChi dataset, it is first manually segmented by thresholding and morphological operation, and then the lung parenchyma is obtained by the same operation as LUNA16. The processing process is shown in Fig. 6. The images from left to right are the original image, binarized image, extracted original mask, complete mask and lung parenchymal image.

### Implementation details

Our model is implemented using the PyTorch deep learning framework and python 3.6 programming language on NVIDIA GeForce RTX 2080Ti GPU. The two proposed networks are trained by SGD optimization algorithm with an initial learning rate of 0.01, a momentum parameter of 0.9 and a weight attenuation coefficient of 0.0001. tenfold cross-validation is used to train the proposed model.

For candidate nodule detection stage, tenfold cross-validation is performed using 888 CT images provided by 10 data subsets of the LUNA16 dataset. Firstly, the images of folds 1–9 are taken as training data to feed into the detection network for training. Then the data of fold 0 are tested on the trained model to get the information on the location, size, and probability of predicted nodules. For each CT image, we only keep the candidate nodules with detection probabilities larger than 0.12. After that, the non-maximum suppression (NMS) with the IoU threshold of 0.1 is adopted to combine the candidate nodules with a high degree of overlap to obtain the final detection result. Finally, the test results are evaluated. Similarly, each fold is taken as the testing set, and the remaining are used as the training sets for a total of 10 training sessions. The average of the 10 evaluation results is taken as the final result to validate the performance of the model. In the experiment, for each fold training and testing, positive samples are augmented via random flipping and scaling between [0.75, 1.25] to alleviate the imbalance problem between positive and negative samples. Each model is trained 100 epochs with learning rate decay strategy. The initial learning rate is 0.01, 0.001 after 50 epochs. The batch size parameter is set to 16 by the limitations of GPU memory. In addition, the 200 CT scans from the validation set of the TianChi dataset are tested on the model trained on the LUNA16 dataset to evaluate the generalization performance of the proposed model.

For false positive reduction stage, the training data comes from the positive and negative candidate samples with labels provided by LUNA dataset, and positive samples are augmented via the same method as candidate nodule detection stage. The 48 × 48 × 48 cubes data are cropped from candidate nodules generated in detection stage and input into the trained 3D deep CNN for classification. During the training process, the Gaussian distribution is used to initialize the weights randomly, and back propagation is used to update the weights. The training batch size is set to 128, the training epoch is set to 100. The learning rate is reduced to 0.001 after 40 epochs, and 0.0001 after 80 epochs.

### Evaluation metrics

In this paper, sensitivity, free-response receiver operating characteristic (FROC), average number of candidate nodules per scan (Avg. candidates/Scan) and competition performance metric (CPM) are used to evaluate the performance of the proposed system. The sensitivity, also known as true positive rate, is an index used in clinical medicine to evaluate the performance of lung nodule detection algorithms. Its formula is as follows:

8 $$Sensitivity = \frac{TP}{{TP + FN}}$$

where $TP$ is the number of all positive detected nodules, $FN$ is the number of positive nodules which are not detected, and $TP + FN$ is the total number of positive nodules. The transverse axis of the FROC curve represents false positives per scan (FPs/Scan), and the longitudinal axis represents sensitivity. Avg. candidates/Scan refers to the average number of candidate nodules detected in each CT. A model with good performance should obtain higher sensitivity under the condition of lower Avg. candidates/Scan. The CPM score is calculated as average sensitivity at the average number of false positives at 0.125, 0.25, 0.5,1, 2, 4, 8 per scan.

### Candidate detection result

In the candidate nodule detection stage, the candidate nodules are obtained through Faster R-CNN based on MAS blocks and U-Net-like network.

#### Ablation studies

To verify the effectiveness of the proposed MSA block, on the LUNA16 dataset, the 3D candidate nodule detection networks based on different residual structures are compared in terms of the sensitivity, CPM score, Avg. candidates/Scan and model size. The experimental results are shown in Table 1. PAO represents pre-activation operation, SE denotes squeeze-and-excitation unit and CQAM indicates convolutional quadruple attention module.

**Table 1:** Performance comparison of different candidate nodule detection network.

Model	Sensitivity	CPM	Avg. candidates/scan	Model size/MB
(A) ResNet	0.938	0.826	33.6	20.54
(B) Res2Net	0.950	0.831	36.5	12.48
(C) Res2Net + PAO	0.953	0.835	32.5	12.48
(D) Res2Net + PAO + SE	0.966	0.852	38.3	12.53
(E) Res2Net + PAO + CQAM	0.963	0.856	27.4	12.49

As can be seen from the experiment (A) (B), when the Res2Net residual block is adopted to replace the basic residual structure as the main component of the network, the sensitivity of nodule detection increased by 1.2%, and the CPM score increased by 0.5% while the model size reduced by nearly 50%. Experiment (C) shows that the addition of pre-activation operation not only reduces the Avg. candidates/Scan but also simultaneously increases the sensitivity and CPM score. To verify the performance of CQAM, experiment (D) and experiment (E) are obtained by integrating the SE block and CQAM based on experiment (C). It can be seen from the comparison results that although the use of the SE block greatly improves the detection sensitivity and CPM score, the Avg. candidates/Scan and the model size also increase significantly. The sensitivity of using CQAM is slightly lower than that of the SE block, but the CPM score is increased by 2.1% compared with experiment (C) with almost no increase in the model size, and Avg. candidates/Scan is reduced by about 5. The above comparison results show that the MSA block proposed in this paper can not only obtain higher sensitivity and CPM score with fewer candidates per scan on average, but also hardly increase the size of the model.

#### Test results

**Figure 7:**
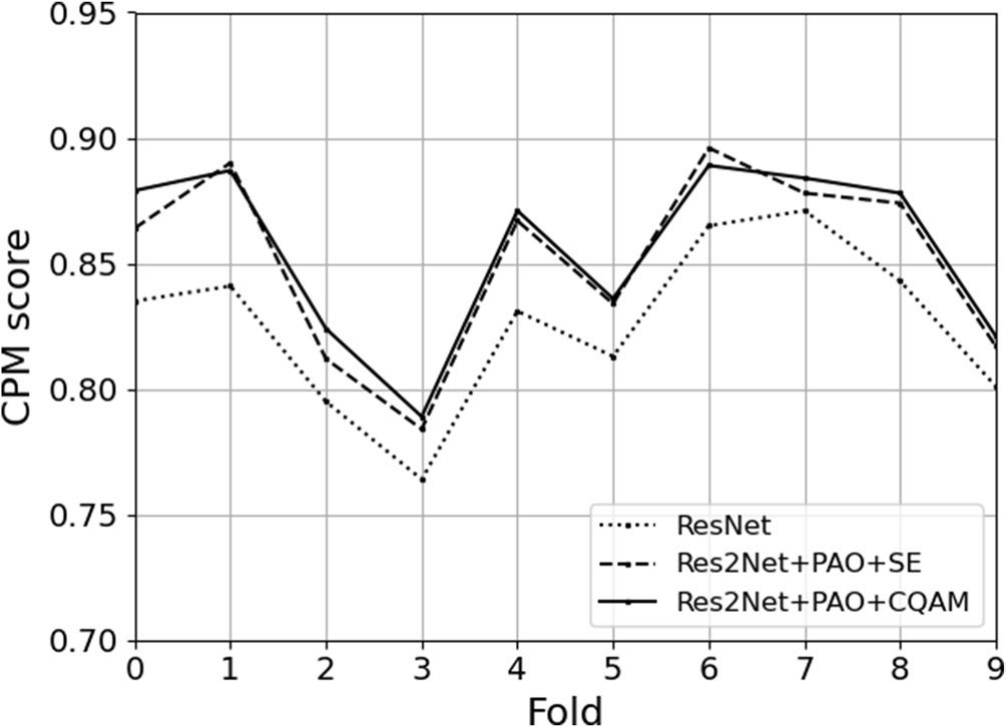
Detection results for each fold on the LUNA16 dataset.

Figure 7 shows the results for each fold in the tenfold cross-validation of the LUNA16 dataset. We compare the detection performance of three different models. One model is based on the basic residual structure of ResNet. The other is based on the MSA block embedded with squeeze-and-excitation unit and pre-activation operation, which can also solve the multi-scale problem. And the last one is the method proposed in this paper, which uses CQAM instead of SE in the MSA block. As can be seen from the figure, the proposed model has higher CPM scores on each fold of the LUNA16 data than the baseline model. And except for the fold 1 and fold 6, which have slightly lower CPM scores than the model using the simple channel attention mechanism, the rest of the folds are better than it.

**Figure 8:**
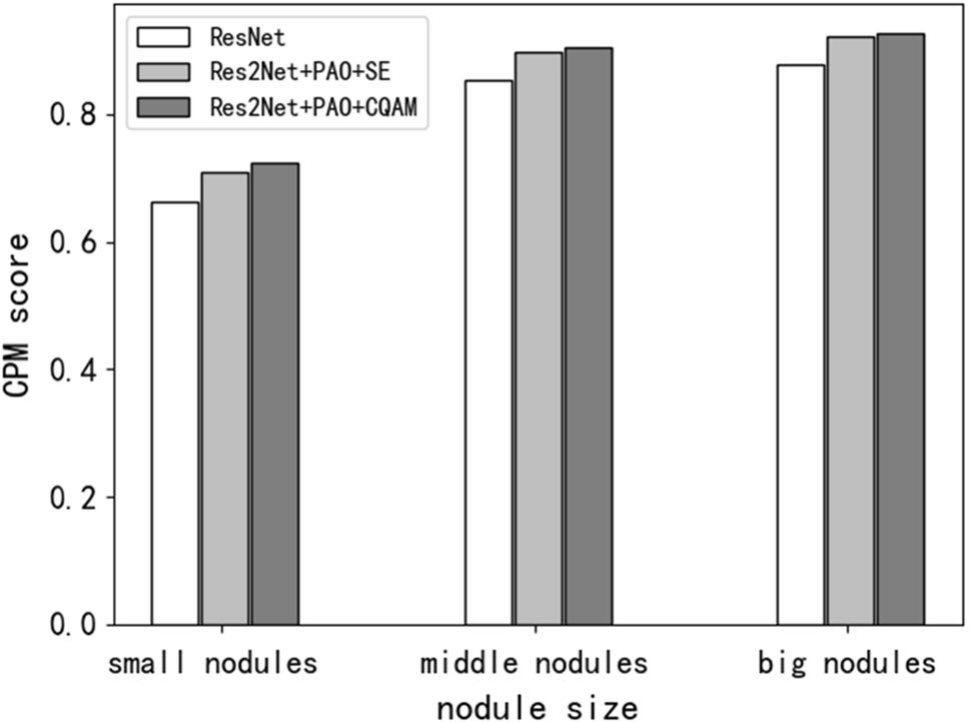
CPM scores of different nodule diameters.

According to the size of the candidate area box set in the previous section, the nodule sizes in the LUNA16 dataset are divided into three categories. They are small nodules (with a diameter of less than 5 mm, accounting for 22.76% in the dataset), medium nodules (with a diameter between 5 and 20 mm, accounting for 73.02%), and large nodules (with a diameter greater than 20 mm, accounting for 4.22%). The relevant information of the predicted nodule is obtained by testing the trained model on the LUNA16 dataset. As shown in Table 2, the first four columns show the serial number and center-of-mass coordinates of the predicted nodules, and the last two columns are the predicted probabilities and diameters. Based on the prediction results, we evaluate the detection effectiveness of the model on pulmonary nodules with different range sizes in the dataset, and the results is shown in Fig. 8. For the medium nodules with the highest proportion in LUNA16, the proposed method achieves a highest CPM score of 0.906. The method in this paper also achieves better detection performance for small nodules, with a CPM score of 0.713.

**Table 2:** The examples of pulmonary nodule detection results on the LUNA16 dataset.

	x	y	z	*p*	d
1	137.10	117.78	− 181.63	0.9986	4.88
2	132.77	16.59	− 130.39	0.9998	10.44
3	82.77	35.36	− 47.24	0.9999	16.95
4	48.49	− 14.38	− 79.54	0.9999	22.78

#### Comparison with other methods

To further evaluate the performance of the proposed nodule candidate detection network, the detection result of this paper on LUNA16 is compared with other existing methods by using the CPM score, and the quantitative results are shown in Table 3. It can be seen from Table 3 that our proposed detection network achieves the highest CPM score of 0.856 and it outperforms state-of-the-art method.

**Table 3:** Comparison of the proposed candidate nodule detection network with other methods.

Methods	Number of FPs/Scan							CPM
	0.125	0.25	0.5	1	2	4	8	
Xie^5^	0.439	0.688	0.796	0.852	0.864	0.864	0.864	0.775
Gu^26^	0.4801	0.6495	0.7920	0.8794	0.9163	0.9293	0.9301	0.7967
Pezeshk^27^	0.637	0.723	0.804	0.865	0.907	0.938	0.952	0.832 ± 0.011
Shi^28^	*	*	*	*	*	*	*	0.8375
Dou^12^	0.659	0.745	0.819	0.865	0.906	0.933	0.946	0.839
Zhu^14^	0.692	0.769	0.824	0.865	0.893	0.917	0.933	0.842
Proposed	0.732	0.774	0.830	0.866	0.917	0.929	0.946	0.856

**Figure 9:**
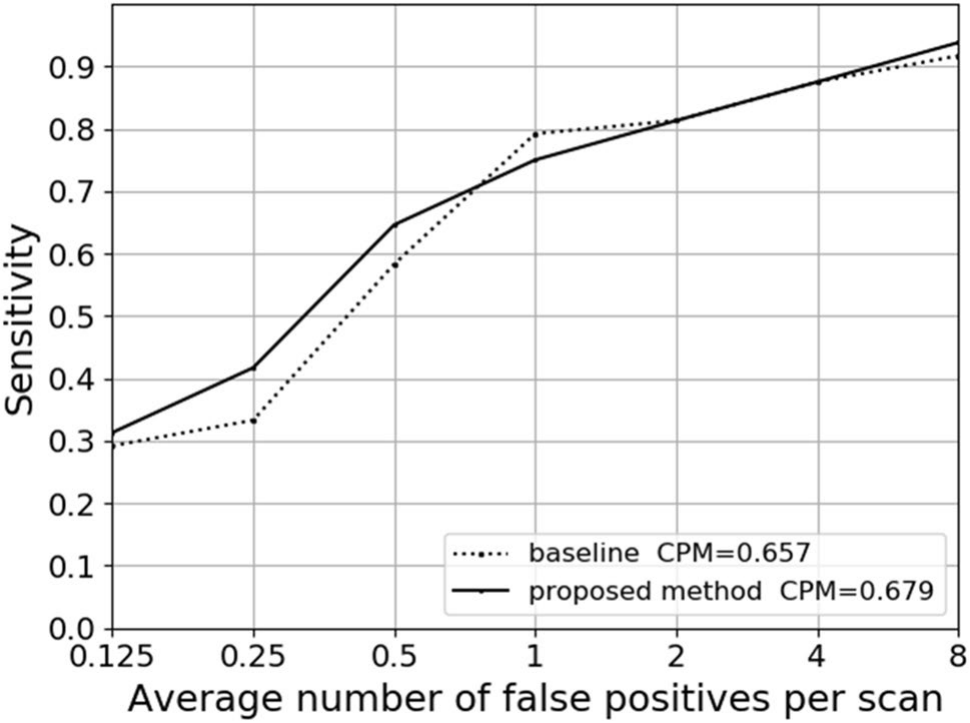
FROC curves obtained by baseline and the proposed methods on TianChi dataset.

Since the training data of the false positive reduction task is not given by the TianChi dataset, the generalization performance of our model is only tested in the candidate nodule detection stage. Firstly, the test experiment is conducted on the TianChi dataset using the trained model by LUNA16. Secondly, the Faster R-CNN based on the basic residual structure is used as the baseline model to test the TianChi dataset. Finally, the two results are compared in the form of the FROC curve shown in Fig. 9. The CPM score (average sensitivity at the false positives as 0.125, 0.25, 0.5, 1, 2, 4, 8) of baseline model is 0.657, while that of the proposed model trained on the LUNA16 dataset is 0.679. It can be seen from the comparison results that the proposed method has better robustness than the baseline method even if it is trained on LUNA16.

### False positive nodule result

The false positive reduction network classifies the candidate nodules obtained in the previous stage, thereby removing false positive samples to make the detection result more accurate. To evaluate the performance of the proposed automatic detection system of pulmonary nodules, we compare our result on LUNA16 with other top methods. As shown in Table 4, it lists the detection sensitivities at 7 different FPs/Scan and the CPM score. Although the automatic detection framework of pulmonary nodules based on 2D CNN proposed by Xie et al. can save training time and storage space, it does not make full use of the 3D information of pulmonary nodules. While among all the methods based on 3D CNN, our proposed detection system achieves the highest CPM score of 0.927, which is 0.4% higher than that of Peng et al. whose model can also extract multi-scale features of nodules by using the Res2Net residual structure. The sensitivities under 1, 2, 4 and 8 FPs/scan are 0.945, 0.953, 0.962 and 0.962, respectively, which are better than the best method presented by Cao et al. Therefore, the method proposed in this paper has superiority and great clinical value.

**Table 4:** Performance comparison of different methods for false positive reduce.

Method	Number of FPs/Scan							CPM
	0.125	0.25	0.5	1	2	4	8	
Xie^5^	0.734	0.744	0.763	0.796	0.824	0.832	0.834	0.790
Khosravan^29^	0.7093	0.8362	0.9208	0.9527	0.9527	0.9527	0.9527	0.8967
Wang^30^	0.788	0.847	0.895	0.934	0.952	0.959	0.963	0.903
Li^31^	0.789	0.847	0.874	0.939	0.964	0.977	0.991	0.912
Qin^32^	*	*	*	*	*	*	*	0.917
Peng^18^	*	*	*	*	*	*	*	0.923
Cao^33^	0.848	0.899	0.925	0.936	0.949	0.957	0.960	0.925
Proposed	0.836	0.898	0.930	0.945	0.953	0.962	0.962	0.927

**Figure 10:**
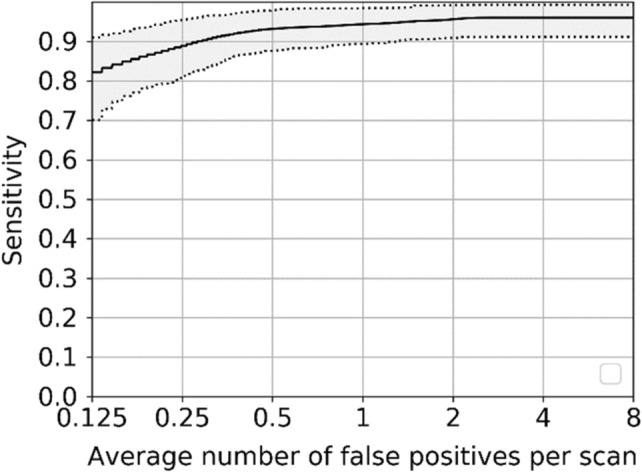
FROC curve of nodule detection system on LUNA16.

Figure 10 uses the FROC curve to show the final test performance of the proposed pulmonary nodule detection system on the LUNA16 dataset. The FROC curve is able to reflect the relationship between the nodules detection rate and the number of false positives per image. The solid line is the interpolated FROC based on true prediction, and the dash lines are lower bound and upper bound FROC for 95% confidence interval using bootstrapping with 1000 bootstraps. When the false positive rate is 0.125, 0.25, 0.5, 1, 2, 4, 8 per case, the sensitivity is 0.836, 0.898, 0.930, 0.945, 0.953, 0.962, 0.962, respectively. And the CPM score is 0.927.

### The visualization of detection results

**Figure 11:**
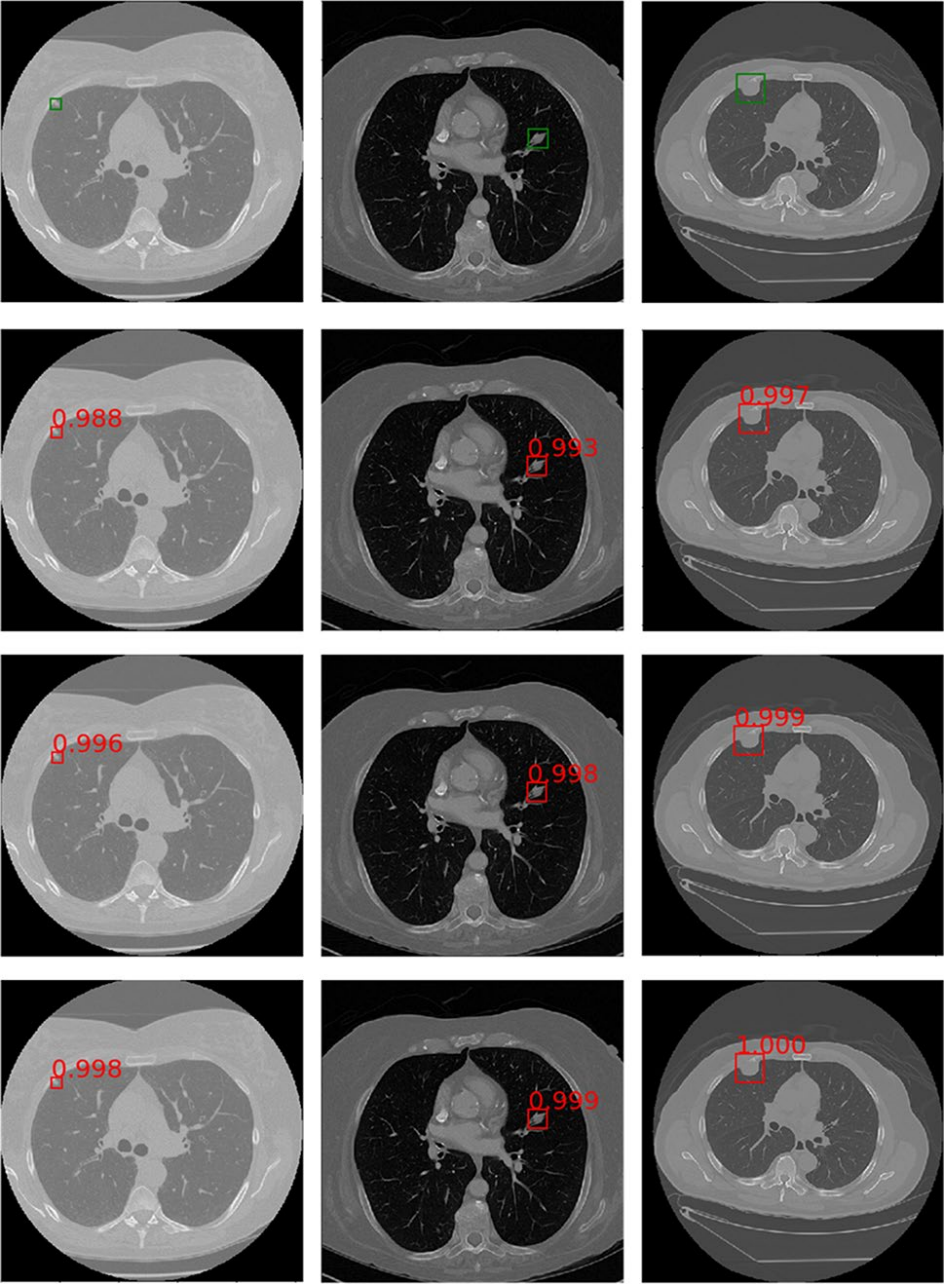
Detection results of nodules with different sizes on LUNA16 dataset.

**Figure 12:**
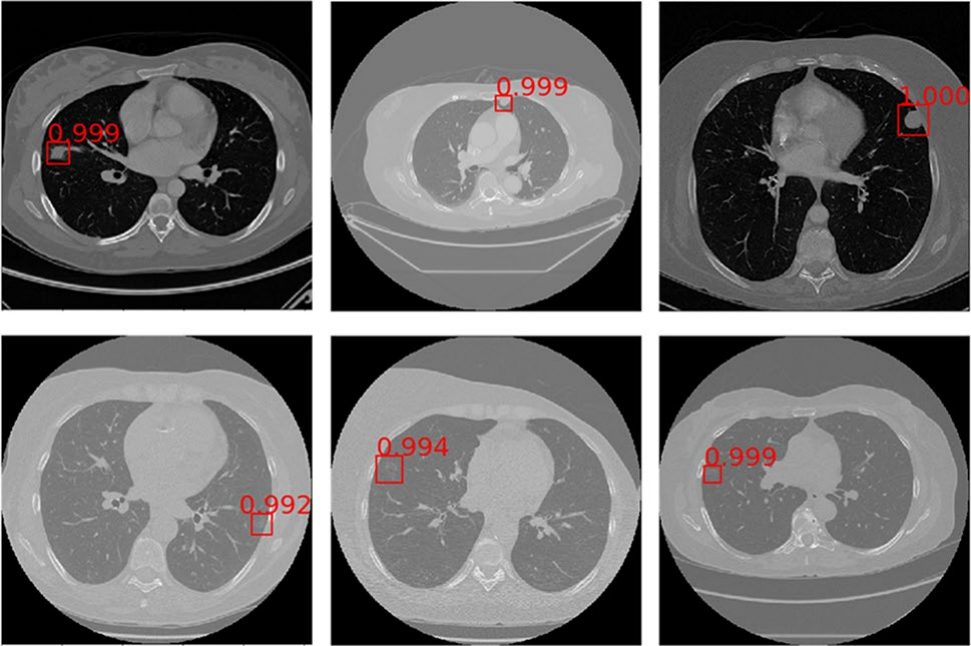
Detection results of nodules with different densities on LUNA16 dataset.

To show the effect of the method proposed by this paper more visually, Fig. 11 lists some detection results of nodules with different sizes on LUNA16. Each row sequentially shows the true nodule labels, the visualization results of the baseline model based on ResNet, the results of the Faster R-CNN detection model based on the MSA block embedded with squeeze-and-excitation unit and pre-activation operation, and that of the proposed method. Each column in turn is small nodules, medium nodules, and large nodules. The detection results of solid nodules and ground glass nodules are shown in Fig. 12. The first row of this figure shows the predicted results of the solid nodule and the second row of the ground glass nodule. The rectangular box in the detection result represents the position of the detected pulmonary nodule. And the number outside the rectangular box represents the confidence level of the predicted nodules.

As can be seen from Figs. 11 and 12, the method in this paper has a high sensitivity in detecting pulmonary nodules of different sizes and densities. As shown in Fig. 11, the baseline model has low detection confidence of small nodules, while the model using squeeze-and-excitation unit has higher confidences in the detection of nodules of different sizes. The method in this paper further improves the detection confidence, which has the best performance. As shown in Fig. 12, the method in this paper achieves high detection confidence for both solid nodules and ground glass nodules.

**Figure 13:**
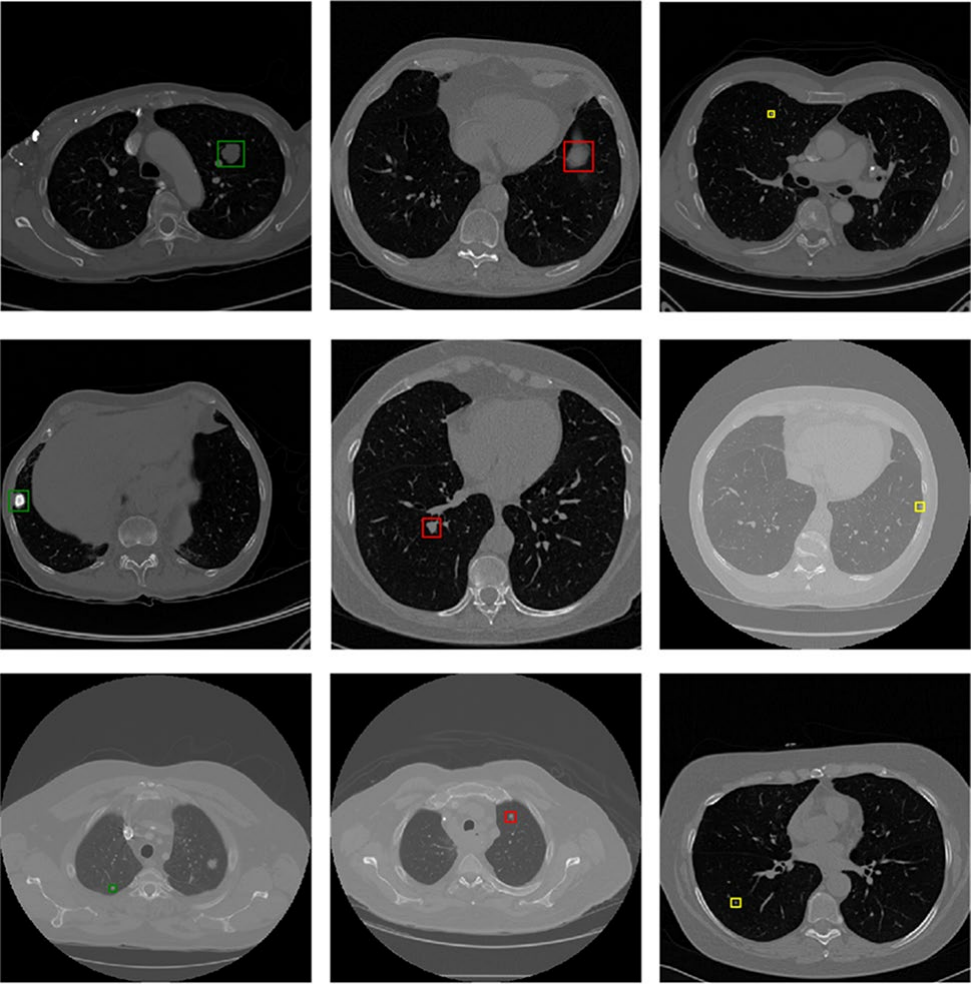
Detection results of true positive nodules, false positive nodules and false negative nodules.

Figure 13 lists the final detection results of the automated pulmonary nodule detection system proposed in this paper on the LUNA16 dataset. The first column images are the true-positive nodules, and the second are the detected false positive nodules that have very similar characteristics to the true nodules. The third column images are the undetected real nodules with extremely small size, which are defined as false negative nodules. The proposed method not only reaches good detection performance for pulmonary nodules with different size and densities but also can accurately detect false-positive nodules.

## Conclusion and discussion

The incidence of lung cancer is increasing year by year. Early detection and treatment can greatly help improve the survival rate of patients. As pulmonary nodules are the early manifestations of lung cancer, the early screening for lung cancer is actually the detection of pulmonary nodules. The model based on 3D CNN is a common method for pulmonary nodule detection, which can fully extract the 3D spatial information of the nodules and has a significant effect on the detection of pulmonary nodules with a specific size. However, we think that the existing 3D detection methods still have room for improvement in making full use of the multi-scale features of nodules and alleviating network over-fitting. To solve these problems, in this paper, an automated pulmonary nodule detection system based on 3D MSA network is proposed, which is composed of two stages, nodule candidate detection and false positive reduction. The proposed MSA block combines multi-scale features not only in a hierarchical manner but also at a granular level, which has stronger multi-scale representation capability. In addition, it also improves the network over-fitting phenomenon by using the pre-activation operation, and makes the network pay more attention to the nodule information useful for the detection task by attention module. For the nodule candidate detection stage, a Faster R-CNN with 3D MSA blocks and a U-net-like encoder-decoder structure is introduced to detect nodules. And For false positive reduction stage, a classifier based on MSA blocks is trained to reduce the false positives generated in the first stage. On LUNA16 dataset, the entire automatic detection system of pulmonary nodules obtains a CPM score of 0.927, which is competitive with other methods. In addition, experiments on the TianChi dataset demonstrated that the proposed model also has good generalization performance.

Although the method presented in this paper can detect most of the nodules, there are still a small number of nodules that are missed. The size of these nodules is extremely small and thus difficult to be detected. Therefore, in the next work, our detection system needs to be optimized in improving the efficiency of small nodules detection. In addition, the scale of the proposed model will be further reduced through some improvements, such as using deep separable convolution instead of basic convolution and so on.

## Data Availability

The used datasets were obtained from publicly open source datasets from: LUNA16 dataset https://luna16.grandchallenge.org/Data/ and TianChi competition dataset https://tianchi.aliyun.com/competition/entrance/231601/information.
